# Pre‐TIPS Liver and Spleen Volumetry Are Not Associated With Liver‐Related Outcomes After TIPS Placement for Refractory Ascites

**DOI:** 10.1111/liv.70691

**Published:** 2026-06-11

**Authors:** José Ursic Bedoya, Mathilde Wagner, Emilie Malézieux, Paul Primard, Hélène Larrue, Sarah Mouri, Charlotte Bouzbib, Marion Soler, Christophe Bureau, Nicolas Molinari, Boris Guiu, Marwa Aboudrar, Calvin Mbenda, Charles Roux, Georges‐Philippe Pageaux, Dominique Thabut, Marika Rudler

**Affiliations:** ^1^ Department of Hepato‐Gastroenterology, Hepatology and Liver Transplantation Unit Saint Eloi Hospital, Univ Montpellier Montpellier France; ^2^ Department of Radiology AP‐HP Sorbonne Université Paris France; ^3^ Department of Hepatology AP‐HP Sorbonne Université Paris France; ^4^ Department of Hepatology University Hospital and Toulouse III—Paul Sabatier University Toulouse France; ^5^ Department of Radiology Saint‐Eloi University Hospital, Univ Montpellier Montpellier France; ^6^ University of Montpellier, University Hospital, IDESP, INSERM, PreMEdical INRIA Montpellier France; ^7^ AP‐HP Sorbonne Université, Department of Interventional Radiology Paris France; ^8^ Brain Liver Pitié‐Salpêtrière Study Group, Sorbonne Université, INSERM UMR_S938, Centre de recherche Saint‐Antoine & Institute of Cardiometabolism and Nutrition (ICAN) Paris France

**Keywords:** cirrhosis decompensation, liver atrophy, liver transplantation, portal hypertension, TIPS complications

## Abstract

**Background & Aims:**

Transjugular intrahepatic portosystemic shunt (TIPS) improves survival in refractory ascites. A careful patient's selection is mandatory as TIPS can lead to complications. Liver volumetry is predictive of outcomes before hepatic surgery, but data on its role before TIPS placement are scarce. We aimed to evaluate whether liver and spleen volume measurements are associated with prognosis after TIPS placement in patients with ascites.

**Methods:**

We analysed data from three French centers, treated with TIPS between 2017 and February 2022. Inclusion criteria encompassed a TIPS placement for refractory or recurrent ascites and availability of cross‐sectional imaging. Exclusion criteria included non‐cirrhotic portal hypertension, other indications for TIPS, hepatocellular carcinoma beyond Milan criteria, and extrahepatic malignancy. Liver and spleen volumes were measured using pre‐TIPS CT or MRI scans. The primary endpoint was 1‐year transplant‐free survival (TFS). Secondary endpoints were overt hepatic encephalopathy (HE), recurrence of ascites, acute variceal bleeding, and jaundice.

**Results:**

The 160 patients were included (median age 60 years, male gender 83.8%, alcohol‐related cirrhosis 58.8%, with active alcohol consumption in 25.6%, Child‐Pugh B cirrhosis in 81.2%, median MELD score was 12). The 1‐year TFS was 60.1%. Multivariate analysis identified serum creatinine (HR = 1.01 95% CI [1.00–1.01], *p* = 0.04), total bilirubin (HR = 1.02 95% CI [1.02–1.04], *p* = 0.004), and portal pressure gradient (HR = 1.09 95% CI [1.01–1.18], *p* = 0.03) as independent factors associated with TFS. Neither liver‐to‐spleen volume ratios (LSVR) (*p* = 0.36) nor liver volume index (*p* = 0.92) were significantly associated with death or LT. Overall, 38.1% of patients developed overt HE after TIPS, with lower platelet count (HR = 1.01 95% CI [1.00–1.01], *p* = 0.04) emerging as an independent predictor. No radiological characteristics were associated with the recurrence of ascites.

**Conclusions:**

In this multicenter study, liver and spleen volumes were not associated with transplant‐free survival or liver‐related outcomes in patients undergoing TIPS for ascites. These findings suggest that liver volumetry should not be a determining factor in patient selection for TIPS placement.

## Lay Summary

Liver volumetry is associated with outcomes after liver surgery. Data are scarce before TIPS placement in patients with cirrhosis and refractory ascites. In this retrospective multicentric study, liver volume was not associated with outcomes after TIPS placement. Liver volumetry should not be a determining factor in the selection of patients for TIPS placement.

**Abbreviations**
AVB: acute variceal bleeding
BNP: Brain natriuretic peptide
CT scan: computed tomography scan
FIPS: Freiburg index prognosis score
HVPG: hepatic venous pressure gradient
LSVR: liver‐to‐spleen volume ratio
LT: liver transplantation
MELD: Model for end stage liver disease
MHE: minimal hepatic encephalopathy
MRI: Magnetic resonance imaging
OHE: overt hepatic encephalopathy
PPG: portal pressure gradient
TFS: Transplant‐free survival
TIPS: transjugular intrahepatic portosystemic shunt

## 1 Introduction

Development of ascites is a turning point in the natural history of cirrhosis. One‐year survival rate is close to 50% [1] in patients with refractory ascites treated with repeated paracentesis. In refractory or recurrent ascites, transjugular intrahepatic portosystemic shunt (TIPS) is associated with a significantly improved survival [1, 2], and is now considered as standard of care [3]. Because TIPS is associated with significant complications (overt hepatic encephalopathy (OHE) and liver failure being the most feared ones), an accurate selection of patients seems to be essential. Besides the classical contraindication to TIPS placement in this setting (liver failure, acute kidney injury, heart failure, severe pulmonary arterial hypertension, the persistence or recurrence of clinical OHE), some authors pointed out several factors associated with a higher risk of OHE, such as a previous episode of HE [1], minimal HE (MHE) [4, 5], sarcopenia [6], age [1], liver failure [1], a low portal pressure gradient (PPG). Several scores have been published to predict outcomes after TIPS: the combination of platelets and serum bilirubin [7] or the Freiburg Index post TIPS survival [8] score, which was first developed in a large German cohort to predict survival after TIPS placement [8]. Besides this, radiological techniques have been developed to predict outcome in liver situations, especially in surgery. Among them, liver volumetry is predictive of liver‐related outcomes after partial hepatectomy [9, 10], alcoholic hepatitis [11] and viral cirrhosis [12]. Indeed, liver volume appears to intuitively reflect liver reserve, and has been used by surgeons for decades to estimate the risk of resection surgery. Hepatic volumetry is used in current practise mainly before carcinologic hepatectomy for primary or secondary liver tumour in order to estimate the volume and therefore the expected function of the liver remaining after surgery. Volumetry is also performed before liver transplantation from a living donor [13]. Nevertheless, data on hepatic volumetry before TIPS placement are scarce, even if a low liver volume may often be considered as a relative contra indication for TIPS placement, especially by some surgeons [14]. Only 3 studies have been conducted in this setting, one of them being published in an abstract form [15, 16]. The results were not really conclusive, the first study suggesting there was no link between hepatic volumetry and prognosis after TIPS placement [15], the second only underlining that the evolution of volumetry (i.e., a decrease in hepatic volume) after TIPS placement was associated with a higher risk of being transplanted and that a ratio hepatic volume/body weight > 20 was associated with a higher transplant free survival (TFS) [16]. Portal hypertension leads to splenomegaly, and a larger spleen volume is associated with worse outcomes in cholestatic [17] or viral liver diseases [18]. Thus, one can hypothesise that liver and spleen volume, as well as liver‐to‐spleen volume ratio (LSVR) might be predictive factors of liver‐related events after TIPS placement. However, data are lacking regarding the predictive value of these parameters in patients treated with TIPS for recurrent or refractory ascites. Therefore, our aim was to evaluate if liver and spleen volume measurements were associated with prognosis after TIPS placement in patients with ascites.

## 2 Methods

### 2.1 Patients

We analysed three French databases including patients with cirrhosis treated with TIPS. These patients were prospectively followed up in 2 centers (Paris, cohort 1 and Toulouse, cohort 3) retrospectively analysed in one center (Montpellier, cohort 2) between 2017 and February 2022. Inclusion criteria were the following: cirrhosis (the diagnosis of cirrhosis was made by unequivocal clinical, biochemical, radiological and/or histological criteria); TIPS placement for refractory or recurrent ascites, i.e., ascites requiring large paracentesis according to referral center; availability of a cross‐sectional imaging (CT or MRI) within 3 months of the TIPS placement. Exclusion criteria were: non cirrhotic portal hypertension; any other indication for TIPS placement, hepatocellular carcinoma (HCC) outside Milan criteria, and extra hepatic malignancy. We intentionally focused on patients undergoing TIPS for refractory or recurrent ascites, as this represents a more homogeneous population than those treated for variceal bleeding. In addition, TIPS for ascites is usually performed as a planned procedure, allowing for systematic pre‐procedural imaging and volumetric assessment. In contrast, TIPS placement for variceal bleeding is most often undertaken as an urgent or rescue intervention, where the decision is made rapidly and without the opportunity for standardised pre‐TIPS imaging. For these reasons, we considered the ascites population the most appropriate to address our study objectives.

### 2.2 Clinical, Biological and Morphological Evaluation Before TIPS Placement

After inclusion, the patient history collection included: history of oesophageal varices (grade), HE, HCC, and portal vein thrombosis (PVT). We detailed for each patient the etiological investigation: viral infection B or C, excessive alcohol consumption (weaned or active), metabolic risk factors (current or past obesity, type 2 diabetes, arterial hypertension, dyslipidaemia), autoimmune hepatitis, biliary disease (primary biliary cholangitis or primary sclerosing cholangitis), Wilson's disease, hemochromatosis and alpha‐1‐anti‐trypsin deficiency. The clinical and biological data collected at inclusion were: sex, age, previous history of decompensation such as ascites, HE, AVB, haemoglobin, platelets, leukocytes, prothrombin time (PT), bilirubin, creatinine, albumin, serum sodium, International Normalised Ratio (INR), aspartate aminotransferase (AST), alanine aminotransferase (ALT), gamma‐glutamyl‐transferase (GGT).

Liver and spleen volumes measurements were measured on the last computed tomography (CT‐scan) or magnetic resonance imaging (MRI) available before TIPS placement. Volume measurements were centralised and carried out by a single senior radiologist (MW) using Syngovia Software (version 8.9, Siemens, Germany) with a semi‐automatic method with manual corrections for the liver volume and a manual segmentation for the spleen volume, blinded to the patient's outcome (Figure S1). For each patient, the liver volume index was defined as the ratio of actual measured liver volume to standardised liver volume. This parameter was calculated using the following validated formula [19]: 191.8 + (18.51 × weight). For each patient, the LSVR was calculated as liver volume divided by spleen volume.

### 2.3 TIPS Procedure

In Paris and Toulouse, TIPS procedure was performed using a 8–10 mm auto‐expandable Viatorr stent‐grafts (Gore) as previously described [20]. In Montpellier, a covered stent (Fluency auto‐expandable, diameter 10 mm) was deployed to cover the whole transhepatic tract and a bare stent (Bard Wallstent, diameter 10 mm) was added (lower end in the portal branch and proximal part at the convergence between the hepatic vein and the vena cava).

### 2.4 Data Recorded During Follow‐Up

Demographic, clinical, and biological data were collected at the time of TIPS placement and until 12 months afterwards. Liver transplantation (LT) and death were collected until date of latest news.

### 2.5 Endpoint and Follow‐Up

Patients were followed up at 1 month, 3 months, and then every 3 months until 1 year after TIPS placement. Decompensation (HE, acute variceal bleeding, ascites, jaundice) and HCC were recorded until 1 year after TIPS placement. The primary endpoint was 1‐year transplant‐free survival (TFS). Secondary endpoints were ascites recurrence, development of HE, jaundice, and AVB. Noteworthy, none of the patients received rifaximin before and after TIPS placement as prophylaxis for overt HE.

### 2.6 Ethics

This research was conducted in accordance with both the Declarations of Helsinki and Istanbul. The study was registered under the French MR‐004 framework which requires that all patients receive written information with the option to oppose the use of their data. This study was approved by the institutional review board of Montpellier University Hospital (IRB‐MTP‐2020‐01‐201 900 310) and by the research ethics committee of Sorbonne University (CER‐2022‐074). All consecutive patients treated with TIPS that were hospitalised between September 2017 and October 2022 were screened for inclusion after collection of their non‐opposition.

### 2.7 Statistical Analyses

Statistical results were presented as median, 1st quartile, and 3rd quartile for quantitative variables. For qualitative variables, the numbers and associated percentages were presented. We used *χ* ^2^ test for categorical variables and Mann Whitney or Student *t*‐test for quantitative variables, according to the normality of the distribution assessed with the Shapiro–Wilk test.

We used the Kaplan–Meier method to estimate survival probabilities and their pointwise 95% confidence intervals. A Cox regression was fitted to account for covariates and to estimate hazard ratios (HR) and their 95% confidence intervals. Survival analyses were performed using the Kaplan–Meier method and compared using the log‐rank test. In addition, competing risk analyses were conducted to account for the presence of mutually exclusive events, namely death and LT. Cumulative incidence functions were estimated for each event. Variables with *p*‐value < 0.1 in univariate analysis were considered for the multivariate model. Data were checked for multicollinearity with the Belsley‐Kuh‐Welsch technique and proportional hazards were checked according to Schoenfeld residuals. The alpha risk was set to 5%. A choice was made by the investigators based on clinical interest to choose between highly collinear variables, and variables with many missing data were excluded. A difference was considered statistically significant when the significance level of the test was < 0.05. Statistical analysis was performed with EasyMedStat (version 3.34.2; www.easymedstat.com).

## 3 Results

### 3.1 Patients

#### 3.1.1 Clinical and Biological Baseline Characteristics of the Whole Cohort

**FIGURE 1:**
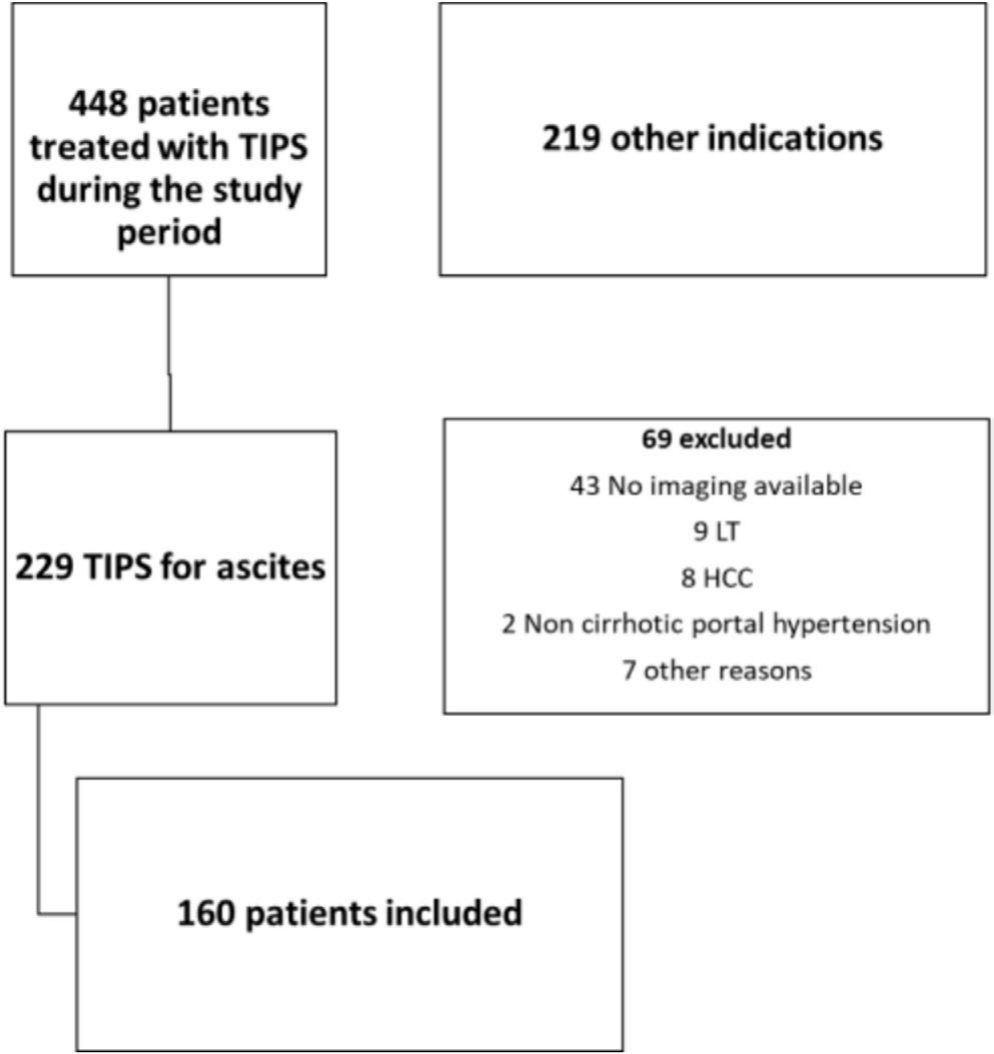
Flow chart of patients included in the study. The 448 procedures were performed during the study period. Among them, 229 were treated with TIPS for ascites, and 69 were excluded: In 43, liver and spleen volumetry measurements could not be performed, 9 had been transplanted previously, 8 had an HCC out of Milan criteria, 2 had non‐cirrhotic portal hypertension, and 7 were excluded for other reasons. Taken together, 160 patients were included. Abbreviations: HCC, hepatocellular carcinoma; LT, liver transplantation; TIPS, transjugular intrahepatic portosystemic shunt.

During the study period, 229 patients were treated with TIPS for ascites and 160 were included in the analysis: 76 from Paris, 62 from Montpellier, and 22 from Toulouse (Figure 1).

The demographic, clinical, and biochemical characteristics of included patients are depicted in Table 1. Most patients were male (83.8%) and had alcohol‐related cirrhosis (ALD) (58.8%), with active alcohol consumption found in 41 patients (25.6%). Most patients were classified as Child‐Pugh B cirrhosis (81.2%); the median MELD score was 12. Table S1 provides baseline characteristics according to the cohort in which patients were included. A previous decompensation was more frequent in cohort 1 when compared to cohorts 2 and 3 (previous HE 23.7% vs. 8.1% and 13.6%, respectively, *p* = 0.044; previous AVB in 27.4% vs. 11.3% and 9.1%, respectively, *p* = 0.025). MELD score tended to be higher in cohort 1, although the difference was not statistically significant.

**TABLE 1:** Baseline characteristics of 160 patients treated with TIPS for refractory or recurrent ascites.

Variable	Data availability	Value	Interquartile range (Q25–Q75)
Age, years	160	60	(53.1–65.0)
Male gender, *n* (%)	160	134 (83.8)	
BMI, kg/m^2^	158	24.6	(21.6–28.0)
Aetiology, *n* (%)			
ALD	160	94 (58.8)	
MASLD		10 (6.2)	
Viral		18 (11.8)	
Met‐ALD		17 (10.5)	
ALD + viral		8 (5.0)	
Other		12 (7.4)	
Liver medical history, *n* (%)			
Hepatic hydrothorax	156	7 (4.5)	
Hepatic encephalopathy	160	26 (16.3)	
Variceal bleeding	157	29 (18.5)	
Hepatocellular carcinoma	160	11 (6.9)	
Paracenteses per month	160	2	(1.5–3.0)
Child‐Pugh score, *n* (%)			
B	160	130 (81.2)	
C		30 (18.8)	
MELD score	160	12.0	(10–15)
Haemoglobin, g/dL	160	10.4	(9.1–12)
Platelets, G/L	160	125	(84–187)
Prothrombin time, %	160	65	(55–76)
INR	155	1.3	(1.2–1.5)
Serum sodium, mmol/L	160	134	(130–137)
Serum creatinine, μmol/L	160	85.5	(66–118)
Albumin, g/L	157	32	(28–35)
Total bilirubin, μmol/L	157	20.3	(13–35)

*Note:* Continuous and categorical variables expressed respectively in median (interquartile range) and *n* (percentages).

Abbreviations: ALD: Alcohol‐related liver disease; BMI: body mass index; INR: International normalised ratio; MASLD: metabolic dysfunction‐associated steatotic liver disease; MELD: model for end‐stage liver disease; TIPS: transjugular intrahepatic portosystemic shunt.

#### 3.1.2 Radiological Characteristics

Median delay between CT scan and TIPS placement was 1 month [0.77–2.48]. Radiological data are depicted in Table 2: liver and spleen volume were acquired thanks to CT scan in 153/160 patients (95.6%) and MRI in 7/160 (4.4%) patients. Median liver and spleen volume was 1527 mL (1214–1848) and 608 mL (413–876), respectively. Table S2 provides radiological data according to cohort 1, 2, and 3. A poor positive correlation was found between standardised liver volume and MELD score (rho = 0.17, *r* ^2^ = 0.016, *p* = 0.03) (Figure S2).

**TABLE 2:** Radiological baseline characteristics of 160 patients treated with TIPS for ascites.

	Data availability	Whole cohort (*n* = 160)	Interquartile range (Q25; Q75)
Cross‐sectional imaging			
CT‐scan	153 (95.6%)		
MRI	7 (4.3%)		
Liver volume (mL)	160	1527	1214–1848
Standardised liver volume (mL)	160	1557	1395–1761
Liver volume index	160	0.99	0.77–1.21
Spleen volume (mL)	159	608	413–876
Liver/spleen volume ratio	159	2.7	1.6–4.0
HVPG (mmHg)	96	16	14–18
PPG (mmHg)	149	7	5.0–8.0

*Note:* Continuous and categorical variables expressed respectively in median (interquartile range) and *n* (percentages).

Abbreviations: CT‐scan, computed tomography; HVPG, hepatic venous pressure gradient; MRI, magnetic resonance imaging; PPG, portal pressure gradient.

### 3.2 Outcomes

Median follow‐up was 26 months (0–81).

#### 3.2.1 One Year Transplant‐Free Survival

**FIGURE 2:**
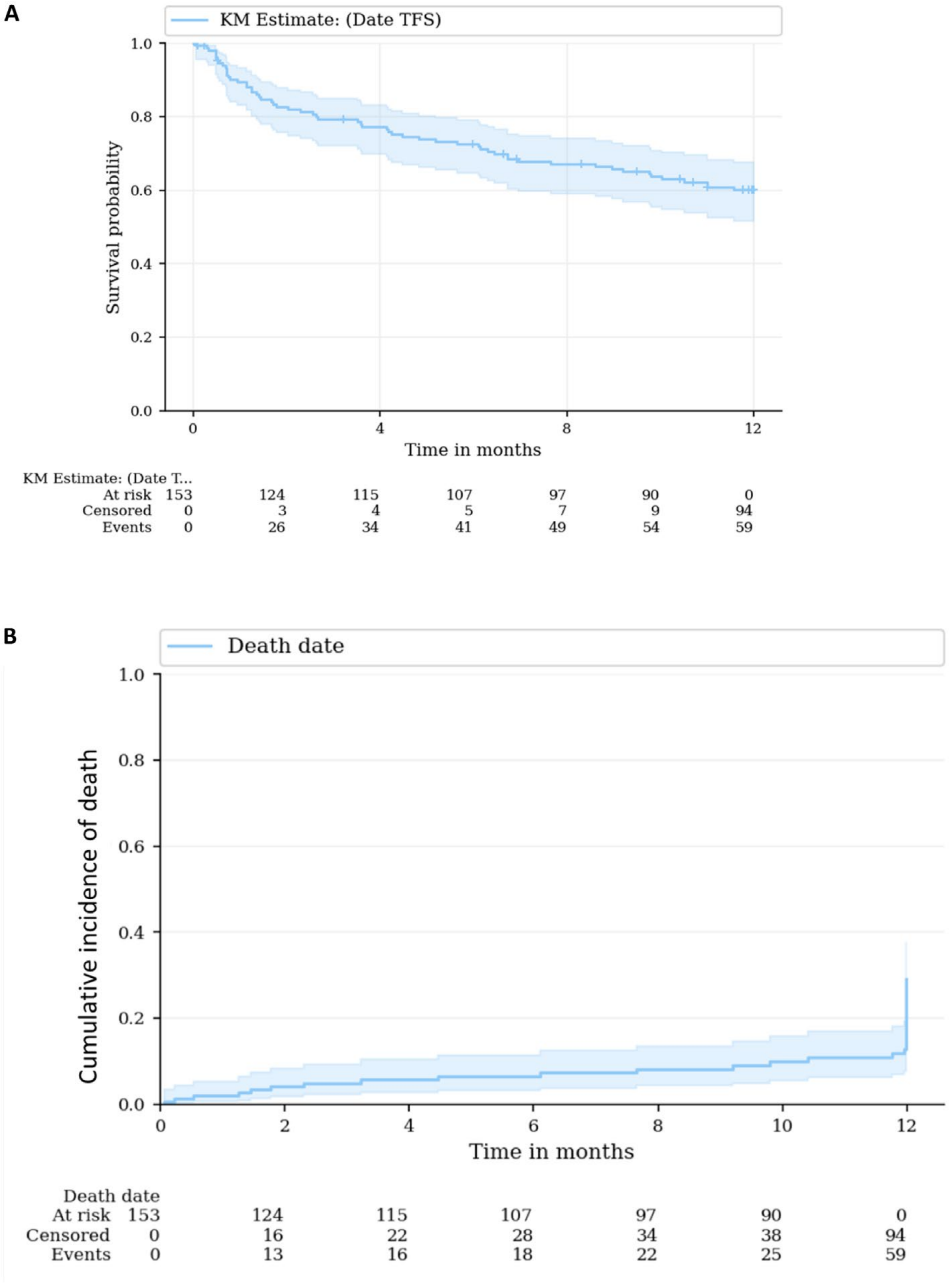
One‐year transplant‐free survival (A) and cumulative incidence of death (B). The 1‐year transplant‐free survival in the whole cohort was 60.1% (95% CI [51.6%–67.5%]). At 12 months, the cumulative incidence of death considering LT as a competing event was 12.7% (7.6%–13.9%).

The 55 patients (34.4%) died, and 39 (24.4%) patients were transplanted. The 1‐year TFS was 60.1% (95% CI: 51.6–67.5) and median TFS was 26.7 months (Figure 2). At 12 months, the cumulative incidence of death considering LT as a competing event was 12.7% (7.6%–13.9%). Causes of death are depicted in Table S3. Radiological data and comparison between the group of living patients and those who died or were transplanted are presented in Table 3. The spleen volume was significantly higher in patients who died or were transplanted (696 vs. 484 mL, *p* = 0.002), whereas liver/spleen volume ratio (LSVR) was significantly lower in the group of patients who died or were transplanted (2.1 vs. 3.3, *p* = 0.002).

**TABLE 3:** Comparison of baseline radiological characteristics in patients alive vs death or transplanted.

	Data availability	Alive (*n* = 66)	Death or transplanted (*n* = 94)	*p*
Liver volume (mL)	160	1588 (1219–1970)	1557 (1217–1779)	0.35
Standardised liver volume (mL)	160	1496 (1321–1761)	1589 (1418–1760)	0.30
Liver volume index	160	1.04 (0.79–1.25)	0.96 (0.74–1.15)	0.08
Spleen volume (mL)	160	484 (372–658)	696 (443–991)	**0.002**
Liver/spleen volume ratio	160	3.3 (2.5–4.3)	2.1 (1.4–3.3)	**0.002**
HVPG (mmHg)	96	16 (13–18)	16 (14–18)	0.97
PPG (mmHg)	149	6 (5.70–6.72)	7 (6.53–7.89)	0.08

*Note:* Bold values are statistically significant (*p* < 0.05).

Abbreviations: HVPG, hepatic venous pressure gradient; PPG, portal pressure gradient.

Factors associated with TFS are depicted in Table 4: in multivariate analysis, independent factors that remained associated with TFS were serum creatinine (HR = 1.01, 95% CI [1.00–1.01] *p* = 0.04), total bilirubin (HR = 1.02, 95% CI [1.00–1.03] *p* = 0.01), and PPG (HR = 1.09, 95% CI [1.01–1.18] *p* = 0.03). Neither LSVR (HR = 0.99, 95% CI [0.83–1.19] *p* = 0.92) nor liver volume index (HR = 0.64, 95% CI [0.25–1.67] *p* = 0.36) were significantly associated with death or LT. In the subgroup of patients with ‘small liver’, i.e., with a LSVR < 1500 mL, LSVR (was not associated with death) (HR = 0.99, 95% CI [0.99–1.00] *p* = 0.48).

**TABLE 4:** Univariate and multivariate analyses of factors associated with transplant‐free survival.

	Univariate analysis			Multivariate analysis		
	Hazard ratio	IC 95%	*p*	Hazard ratio	IC 95%	*p*
Gender						
Male	1					
Female	0.65	[0.33–1.29]	0.22			
Age	0.99	[0.96–1.01]	0.30			
Total bilirubin (each increase of 1 unit)	1.02	[1.01–1.03]	< 0.001	1.02	[1.00–1.03]	**0.004**
Serum creatinine	1	[1–1.01]	0.06	1.01	[1.00–1.01]	**0.04**
Albumin	0.99	[0.96–1.04]	0.95			
Platelets count	0.99	[0.99–1.00]	0.002	0.99	[0.99–1.00]	0.64
Child‐Pugh score	1.13	[0.95–1.34]	0.16			
History of hepatic encephalopathy	1.7	[1.03–2.83]	0.04	1.46	[0.85–2.50]	0.17
ALD	0.83	[0.51–1.36]	0.44			
Liver volume index	0.54	[0.28–1.08]	0.08	0.64	[0.25–1.67]	0.36
Liver/spleen volume ratio	0.85	[0.75–0.98]	0.02	0.99	[0.83–1.19]	0.92
PPG	1.09	[1.01–1.17]	0.02	1.09	[1.01–1.18]	**0.03**

*Note:* Bold values are statistically significant (*p* < 0.05).

Abbreviations: ALD, alcohol‐related liver disease; PPG, portal pressure gradient.

#### 3.2.2 Development of OHE After TIPS

**FIGURE 3:**
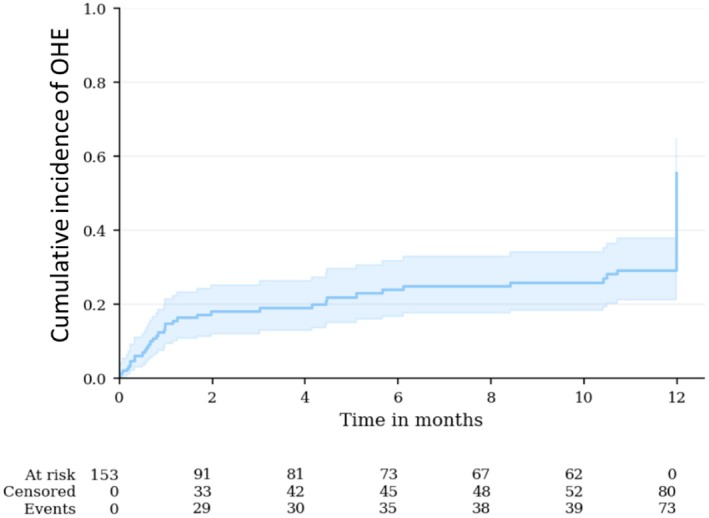
Cumulative incidence of OHE during follow‐up. At 12 months, the cumulative incidence of OHE considering LT and death as a competing event was 29.3% (21.2%–37.8%).

The 61 patients developed at least one episode of OHE after TIPS (38.1%) in a median delay of 19 days (Figure 3). At 12 months, the cumulative incidence of OHE considering LT and death as a competing event was 29.3% (21.2%–37.8%). In univariate analysis, an older age (*p* = 0.04), a lower platelet count (*p* = 0.008), a lower liver volume index (*p* = 0.008), and a lower LSVR (*p* = 0.04) were associated with HE occurrence. In multivariate analysis, the only independent factor that remained associated with the occurrence of HE after TIPS was platelet count (HR = 0.99, 95% CI [0.99–1.00] *p* = 0.048) (Table S4). Neither LSVR (HR = 1.05, 95% CI [0.86–1.28] *p* = 0.61) nor liver volume index (HR = 0.46, 95% CI [0.17–1.26] *p* = 0.13) were significantly associated with OHE.

#### 3.2.3 Development of Ascites After TIPS

**FIGURE 4:**
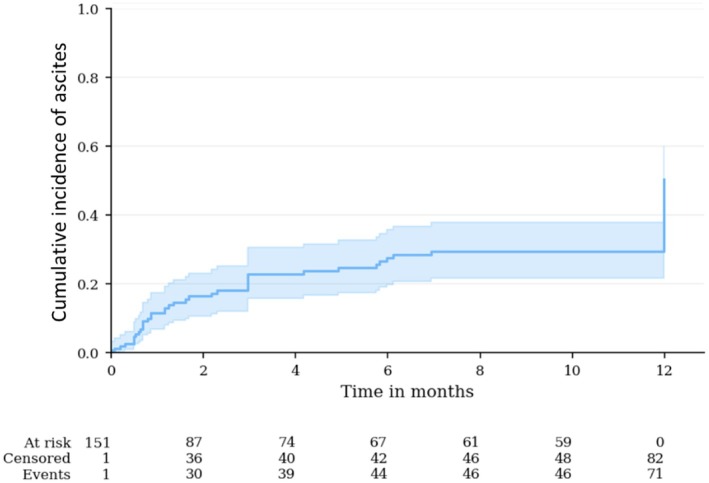
Cumulative incidence of ascites during follow‐up. At 12 months, the cumulative incidence of ascites considering LT and death as a competing event was 50.5% (40.2%–59.8%).

The 60 patients (37.5%) had recurrence of ascites after TIPS needing diuretics and/or paracenteses in a median delay of 30 days, including 13 (8.0%) who met the criteria for refractory ascites (Figure 4). At 12 months, the cumulative incidence of ascites considering LT and death as a competing event was 50.5% (40.2%–59.8%). None of the radiological characteristics were associated with further development of ascites. We found no factor predictive of post‐TIPS ascites recurrence (Table S5).

#### 3.2.4 Development of Jaundice and AVB

Thirty‐four patients (21.3%) had at least one episode of jaundice following TIPS placement, whereas three patients (1.9%) developed an AVB. In multivariate analysis, MELD score (OR = 1.15, [1.04; 1.26], *p* = 0.006) was associated with higher rates of post‐TIPS jaundice (Table S6). Neither LSVR (HR = 0.98, 95% CI [0.78–1.23], *p* = 0.88) nor liver volume index (HR = 0.28, 95% CI [0.39–4.21], *p* = 0.69) were significantly associated with further development of jaundice (Table S7).

#### 3.2.5 Post Hoc Analysis

In a post hoc analysis, we evaluated the influence of LSVR in the subgroup of patients with LSVR < 1500 mL: LSVR was not associated with death (HR = 0.99, 95% CI [0.99–1.00], *p* = 0.48) or overt HE (HR = 1 [0.99–1.01], *p* = 0.29). Additionally, LSVR was not associated with death (HR = 1, 95% CI [0.99–1.00], *p* = 0.61) or overt HE (HR = 1, 95% CI [1.00–1.00], *p* = 0.07) in the subgroup of patients without previous overt HE or AVB (Table S8a–d).

## 4 Discussion

In this study, which included patients from 3 different centres, we found that neither liver volume nor liver/spleen ratio was associated with TFS in patients treated with TIPS for refractory or recurrent ascites. Notably, none of the radiological parameters were independently associated with liver outcomes after TIPS, such as HE or recurrent ascites. Taken together, these data suggest that liver volume assessment should not be considered prior to TIPS placement since functional reserve, not anatomical volume, dictates post‐TIPS outcomes. This finding is important as ‘reduced liver volume’ is currently considered a contra‐indication for TIPS placement by some physicians, although not supported by a very recent review of the literature [21] or clinical practise guidelines [22].

The pathophysiological hypothesis of the predictive value of liver volumetry after TIPS comes from surgical studies, mostly in the setting of hepatocellular carcinoma. There is a correlation between the residual hepatic volume after resection and the risk of further hepatic failure [23]. Indeed, in cirrhosis, a 50% residual hepatic volume after resection is usually considered safe regarding the risk of further development of decompensation after liver surgery. In another context, and outside TIPS placement, Patel and colleagues [24] evaluated the utility of liver and spleen volume measurement in a large cohort of patients with cirrhosis for the prediction of liver outcomes. The authors observed an association between HE and a lower liver volume. Moreover, patients who underwent LT during follow‐up had a higher spleen volume. It seemed rational to evaluate the liver/spleen volume ratio in the context of TIPS placement, a situation in which patients may develop HE and liver failure.

Many studies described factors associated with worse outcomes after TIPS placement: the combination of platelets and bilirubin [7], the FIPS score [8], the MELD or Child‐Pugh scores [1]. It should be underlined that these biological data or scores are not specific for further liver decompensation after TIPS but are associated with a poor prognosis in decompensated cirrhosis in general. For this reason, we strongly emphasise that the decision for TIPS placement should be discussed together with liver transplantation, as the indication for TIPS placement is usually an indication for liver transplantation, and the decision should be made after a multidisciplinary discussion [14]. A clear demonstration of the impact of liver atrophy on prognosis after TIPS placement would come from a large randomised controlled trial comparing TIPS and standard of care in the management of portal‐related complications showing that atrophy has an impact in patients treated with TIPS but no impact in patients receiving standard of care treatment.

Our results confirm the prognostic value of MELD and FIPS components such as bilirubin and serum creatinine. Interestingly, post‐procedure PPG also appeared statistically associated with worse outcomes after TIPS placement for recurrent and refractory ascites. This parameter is usually obtained at the end of the procedure and has only informative value. It is unclear whether modulating this parameter would result in different outcomes. This would require larger scale, specifically designed studies.

Previous studies have assessed liver volumetry in the context of TIPS placement. Lopera et al. [15] analysed the association between post‐TIPS adverse events and raw liver volume (without standardisation to the weight or body surface area of the patient) in a cohort of 80 patients receiving elective TIPS placement, mostly for ascites. The authors did not find any association between liver volume and adverse outcomes after TIPS placement, although they noticed a significantly reduced liver volume in patients receiving LT compared to those who did not need LT. More recently, Schindler et al. [16] investigated the relationship between liver volume adjusted to body weight and TFS after TIPS placement. Interestingly, they observed that liver volume decreased over time, more rapidly in smaller livers. They also noticed that lower total liver volume/weight ratios before TIPS were associated with shorter TFS. It is worth noting that none of these studies simultaneously examined liver and spleen volume.

Our study has several limits, the first one being linked to its retrospective nature. We acknowledge that a selection bias could not be excluded as 30% of our patients for whom TIPS was performed during the study period were excluded, mainly because of the absence of CT scan or MRI performed in due time. Moreover, follow‐up data, especially hepatic volume reduction after TIPS placement, could not be evaluated in our cohort of patients. Indeed, this parameter was found to be predictive of the need for liver transplantation in the study from Schindler et al. [16]. However, even if we had confirmed this result, this parameter would not have helped in decision making for TIPS placement, as the liver volume evolution is by definition calculated after TIPS placement. Nonetheless, liver volume is not considered before TIPS placement [21, 22].

Moreover, our cohort is made up of highly selected patients, which results in the lack of prognostic value of parameters such as age or past hepatic encephalopathy. One could argue that this selection has hence excluded patients with the most atrophic livers. If this is the case, our results at least have the merit of showing that it does not add any information to the usual evaluation of liver function before TIPS placement. Finally, we used a single experienced radiologist to perform volume measurements to improve consistency; however, this precludes assessment of inter‐observer variability and reproducibility.

In conclusion, in our study, liver or spleen volume was neither associated with TFS nor with liver‐related outcomes in patients undergoing TIPS for ascites.

## Author Contributions

Design: José Ursic Bedoya, Mathilde Wagner, Georges‐Philippe Pageaux, Dominique Thabut, Marika Rudler. Data acquisition: José Ursic Bedoya, Mathilde Wagner, Emilie Malézieux, Paul Primard, Hélène Larrue, Sarah Mouri, Charlotte Bouzbib, Marwa Aboudrar, Calvin Mbenda, Charles Roux, Marika Rudler. Data analysis and interpretation: José Ursic Bedoya, Marion Soler, Nicolas Molinari, Marika Rudler. Drafting: José Ursic Bedoya, Marika Rudler. Critical revision: Charlotte Bouzbib, Dominique Thabut, Boris Guiu, Georges‐Philippe Pageaux, Dominique Thabut. All authors approved the final version of the article.

## Funding

The authors have nothing to report.

## Conflicts of Interest

Marika Rudler speaker for Gore and Abbvie. José Ursic Bedoya received travel and congress fees from Gilead, Abbvie, Chiesi, and Astellas. Charles Roux speaker for Gore and Siemens Healthineers. The other authors declare no conflicts of interest.

## Supporting information


**Table S1.** Baseline characteristics of 43 patients who were not included in the study.
**Table S2.** Baseline characteristics of the patients according to the cohort in which patients were included.
**Table S3.** Baseline radiological characteristics in cohort 1, 2 and 3.
**Table S4.** Causes of death.
**Table S5.** Univariate and multivariate analyses of variables associated with development of hepatic encephalopathy after TIPS.
**Table S6.** Univariate and multivariate analyses of variables associated with ascites recurrence in patients with TIPS placement for refractory ascites.
**Table S7.** Independent factors associated with further development of jaundice in multivariate analysis.
**Table S8a.** Univariate and multivariate analysis of factors associated with death in cohort 1.
**Table S8b.** Univariate and multivariate analysis of factors associated with overt HE in cohort 1.
**Table S8c.** Univariate and multivariate analysis of factors associated with death in cohort 2.
**Table S8d.** Univariate and multivariate analysis of factors associated with overt HE in cohort 2.
**Figure S1.** Acquisition of liver volume. (a) Automatic acquisition of liver contouring; (b), manual correction; (c), determination of liver volume.
**Figure S2.** Correlation between standardised liver volume and MELD score. A poor positive correlation was found between standardised liver volume and MELD score (rho = 0.17, *r* ^2^ = 0.016, *p* = 0.03).

## Data Availability

The data that support the findings of this study are available on request from the corresponding author. The data are not publicly available due to privacy or ethical restrictions.
