# Supplementary material for: Pre‐TIPS Liver and Spleen Volumetry Are Not Associated With Liver‐Related Outcomes After TIPS Placement for Refractory Ascites
Source: Liver Int. 2026 Jun 11;46(7):e70691. doi: 10.1111/liv.70691 (PMC13255523; doi:10.1111/liv.70691)
Supplement: Supplementary file 1 — Table S1. Baseline characteristics of 43 patients who were not included in the study. Table S2. Baseline characteristics of the patients according to the cohort in which patients were included. Table S3. Baseline radiological characteristics in cohort 1, 2 and 3. Table S4. Causes of death. Table S5. Univariate and multivariate analyses of variables associated with development of hepatic encephalopathy after TIPS. Table S6. Univariate and multivariate analyses of variables associated with ascites recurrence in patients with TIPS placement for refractory ascites. Table S7. Independent factors associated with further development of jaundice in multivariate analysis. Table S8a. Univariate and multivariate analysis of factors associated with death in cohort 1. Table S8b. Univariate and multivariate analysis of factors associated with overt HE in cohort 1. Table S8c. Univariate and multivariate analysis of factors associated with death in cohort 2. Table S8d. Univariate and multivariate analysis of factors associated with overt HE in cohort 2. Figure S1. Acquisition of liver volume. (a) Automatic acquisition of liver contouring; (b), manual correction; (c), determination of liver volume. Figure S2. Correlation between standardised liver volume and MELD score. A poor positive correlation was found between standardised liver volume and MELD score (rho = 0.17, r 2 = 0.016, p = 0.03). [file LIV-46-0-s001.docx]

# **Supplementary material**

**Title: Pre-TIPS Liver and Spleen Volumetry are Not Associated With Liver-related Outcomes After TIPS Placement for Refractory Ascites**

Short title: Volumetry is not useful before TIPS for refractory ascites

**Table of contents**

Supplementary Table 1: page 2

Supplementary Table 2: page 3

Supplementary Table 3: page 5

Supplementary Table 4: page 6

Supplementary Table 5: page 7

Supplementary Table 6: page 8

Supplementary Table 7: page 8

Supplementary Table 8: page 9

Supplementary Figure 1: page 11

Supplementary Figure 2: page 11

| Variable | Excluded patients  N=43 | Patients included in the study N=160 | P value |
| --- | --- | --- | --- |
| Age, years | 59 (53.0-64.0) | 60 (51.3-65.0) | 0.31 |
| Male gender, n (%) | 37 (86.0) | 134 (83.8) | 0.82 |
| BMI, kg/m² | 25.2 (21.6 -29.0) | 24.6 (21.6 -28.0) | 0.47 |
| Aetiology, n (%)  ALD  MASLD  Viral  Met-ALD  ALD + viral  Other | 17 (32.5)  4 (9.3)  4 (9.3)  8 (18.6)  3 (7.0)  7 (16.2) | 94 (58.8)  10 (6.2)  18 (11.8)  17 (10.5)  8 (5.0)  12 (7.4) | 0.20 |
| Liver medical history, n (%)  Hepatic hydrothorax  Hepatic encephalopathy  Variceal bleeding  Hepatocellular carcinoma | 2 (4.6)  9 (20.9)  8 (18.6)  1 (2.3) | 7 (4.5)  26 (16.3)  29 (18.5)  11 (6.9) | 0.87  0.26  0.99  0.47 |
| Paracenteses per month | 2 (1.4-3.1) | 2 (1.5-3.0) | 0.82 |
| Child-Pugh score, n (%)  B  C | 38 (90.0)  5 (10.0) | 130 (81.2)  30 (18.8) | 0.15 |
| MELD score | 11.5 (10-14) | 12 (10-15) | 0.23 |
| Haemoglobin, g/dL | 10.8 (9.3-12.6) | 10.4 (9.1-12.0) | 0.30 |
| Platelets, G/L | 130 (80-176) | 125 (84-187) | 0.61 |
| PT, % | 65 (56-73) | 65 (55-76) | 0.99 |
| INR | 1.3 (1.2-1.5) | 1.3 (1.2-1.5) | 0.77 |
| Serum sodium, mmol/L | 134 (131-137) | 134 (131-137) | 0.97 |
| Serum creatinine, µmol/L | 90 (71-110) | 85.5 (66-118) | 0.72 |
| Albumin, g/L | 32 (27-34) | 32 (28-35) | 0.16 |
| Total bilirubin, µmol/L | 21 (13-36) | 20.3 (13-35) | 0.37 |

**Supplementary Table 1: baseline characteristics of 43 patients who were not included in the study** *Abbreviations: BMI, body mass index; ALD, alcohol-related liver disease; MASLD, metabolic dysfunction-associated steatotic liver disease; MetALD, metabolic dysfunction-associated alcohol-associated liver disease; MELD, model for end stage liver disease; PT, prothrombin time ratio; INR, international normalized ratio;*

## **Supplementary Table 2: baseline characteristics of the patients according to the cohort in which patients were included**

| Variable | Cohort 1  N = 76 | Cohort 2  N = 62 | Cohort 3  N = 22 | p-Value |
| --- | --- | --- | --- | --- |
| **Age (years)** | 58.8 (52.3-62.1) | 60.1 (57.4-63.8) | 62.4 (52.7-66.9) | 0.11 |
| **BMI (kg/m2)** | 25 (23-28) | 25 (21-28) | 23 (20-27) | 0.19 |
| **Hemoglobin (g/dL)** | 10.0 (8.8-11.8) | 10.4 (9.4-13.1)) | 10.9 (9.3-12.2) | 0.26 |
| **Platelets count (G/L)** | 115 (77-159) | 145 (101-191) | 155 (95-212) | 0.05 |
| **PT (%)** | 64 (54-74) | 67 (60-82) | 64 (55-75) | 0.09 |
| **INR** | 1.3 (1.2-1.5) | 1.3 (1.1-1.5) | 1.3 (1.2-1.5) | 0.29 |
| **Serum sodium** | 134 (129-138) | 136 (132-138) | 133 (129-135) | 0.05 |
| **Creatinine** | 81 (66-112) | 87 (62-120) | 91 (67-133) | 0.53 |
| **Albumin** | 32 (27-35) | 33 (31-36) | 30 (27-33) | **0.03** |
| **Bilirubin** | 22 (13-36) | 19 (12-36) | 19 (15-25) | 0.47 |
| **Previous HE** | 18 (23.7%) | 5 (8.1%) | 3 (13.6%) | **0.04** |
| **Previous AVB** | 20 (27.4%) | 7 (11.3%) | 2 (9.1%) | **0.03** |
| **Previous HCC** | 4 (5.3%) | 4 (6.4%) | 3 (13.6%) | 0.39 |
| **Number of paracenteses (/month)** | 3 (2-4) | 2 (1-3) | 2 (1-3) | **0.03** |
| **Child-Pugh score** | 8 (8-10) | 8 (8-9) | 8 (8-9) | 0.05 |
| **MELD score** | 13 (11-16) | 12 (9-15) | 11 (10-13) | 0.08 |
| **HVPG** | 15 (13-21) | 16 (14-17) | 16 (15-18) | 0.74 |
| **PPG** | 7 (5-8) | 6 (5-8) | 6 (5-7) | 0.42 |
|  |  |  |  |  |
|  |  |  |  |  |

*Abbreviations: BMI, body mass index; PT, prothrombin time ratio; INR, international normalized ratio; HE, hepatic encephalopathy; AVB, acute variceal bleeding; HCC, hepatocellular carcinoma; MELD, model for end stage liver disease; HVPG, hepatic venous pressure gradient; PPG, portal pressure gradient*

## **Supplementary Table 3: baseline radiological characteristics in cohort 1, 2 and 3**

| Variable | Cohort 1  N = 76 | Cohort 2  N = 62 | Cohort 3  N = 22 | p-Value |
| --- | --- | --- | --- | --- |
| **Liver volume (mL)** | 1453 (1220-1734) | 1451 (1166-1857) | 1659(1432-2058) | 0.17 |
| **Spleen volume (mL)** | 673 (417-985) | 530 (389-795) | 496 (425-798) | 0.08 |
| **Standardized liver volume (mL)** | 1589 (1409-1765) | 1562 (1335-1710) | 1478 (1372-1774) | 0.63 |
| **Liver volume index** | 0.977 (0.739-1.110) | 0.966 (0.777-1.230) | 1.200 (0.988-1.320) | **0.02** |
| **Liver to spleen volume ratio** | 2.27 (1.43-3.54) | 3.10 (1.86-4.29) | 3.26 (2.58-4.31) | **0.04** |

## **Supplementary Table 4: causes of death**

| **Causes** | **N** | **%** |
| --- | --- | --- |
| Liver related | 36 | 65% |
| Malignancy | 5 | 9% |
| Others  Unknown | 9  5 | 16%  9% |
| **Total** | **55** | **100%** |
|  |  |  |
|  |  |  |

## **Supplementary Table 5: Univariate and multivariate analyses of variables associated with development of hepatic encephalopathy after TIPS**

|  | Univariate analysis | | | Multivariate analysis | | |
| --- | --- | --- | --- | --- | --- | --- |
|  | Hazard ratio | IC 95% | p | Hazard ratio | IC 95% | p |
| Gender  Male  Female | 1  1.03 | [0.51-2.1] | 0.92 |  |  |  |
| Age | 1.03 | [1.00-1.07] | 0.04 | 1.02 | [0.99-1.07] | 0.12 |
| Total bilirubin (each increase of 1 unit)^#^ |  |  |  |  |  |  |
| Serum creatinine^#^ |  |  |  |  |  |  |
| History of hepatic encephalopathy | 1.32 | [0.69-2.55] | 0.40 |  |  |  |
| Albumin | 0.97 | [0.92-1.02] | 0.26 |  |  |  |
| Platelets count | 0.99 | [0.99-1.00] | 0.007 | 0.99 | [0.99-1.00] | **0.04** |
| Serum sodium ^#^ |  |  |  |  |  |  |
| Liver volume index | 0.29 | [0.11-0.72] | 0.008 | 0.46 | [0.17-1.26] | 0.13 |
| Liver / spleen volume ratio | 0.86 | [0.74-0.99] | 0.04 | 1.05 | [0.86-1.28] | 0.61 |
| PPG | 1.03 | [0.94-1.12] | 0.55 |  |  |  |
|  |  |  |  |  |  |  |

^#^ Not analysed due to lack of log-linearity

*PPG, portal pressure gradient*

## **Supplementary Table 6: Univariate and multivariate analyses of variables associated with ascites recurrence in patients with TIPS placement for refractory ascites.**

|  | Univariate analysis | | | Multivariate analysis | | |
| --- | --- | --- | --- | --- | --- | --- |
|  | Hazard ratio | IC 95% | p | Hazard ratio | IC 95% | p |
| Gender  Male  Female | 1  1.27 | [0.64-2.53] | 0.49 |  |  |  |
| Age | 0.98 | [0.95-1.02] | 0.32 |  |  |  |
| Total bilirubin (each increase of 1 unit) | 0.99 | [0.98-1.01] | 0.45 |  |  |  |
| Serum creatinine | 1 | [1.00-1.01] | 0.22 |  |  |  |
| Albumin | 1.03 | [0.97-1.09] | 0.32 |  |  |  |
| Platelets count | 0.99 | [0.99-1] | 0.10 | 0.99 | [0.99-1.00] | 0.41 |
| History of hepatic encephalopathy | 0.48 | [0.17-1.33] | 0.16 |  |  |  |
| Liver volume index | 1.09 | [0.44-2.72] | 0.85 |  |  |  |
| Liver / spleen volume ratio | 0.884 | [0.76-1.03] | 0.10 | 0.93 | [0.77-1.12] | 0.44 |
| PPG | 0.984 | [0.89-1.09] | 0.76 |  |  |  |

*PPG, portal pressure gradient*

## **Supplementary Table 7: Independent factors associated with further development of jaundice in multivariate analysis**

| Variable | Odds ratio | p-value |
| --- | --- | --- |
| Age | 0.98 [0.93;1.03] | 0.45 |
| Baseline MELD score | 1.15 [1.04;1.26] | **0.006** |
| Liver/spleen volume ratio  Liver volume index | 0.98 [0.78;1.23]  1.28 [0.39-4.21] | 0.88  0.80 |

**Supplementary Table 8 a: Univariate and multivariate analysis of factors associated with death in cohort 1**

|  | Univariate analysis | | | Multivariate analysis | | |
| --- | --- | --- | --- | --- | --- | --- |
|  | Hazard ratio | IC 95% | p | Hazard ratio | IC 95% | p |
| Gender  Male  Female | 1  0.65 | [0.33-1.29] | 0.22 |  |  |  |
| Age | 0.99 | [0.94-1.04] | 0.62 |  |  |  |
| Total bilirubin (each increase of 1 unit) | 1.01 | [0.99-1.03] | 0.35 |  |  |  |
| Serum creatinine | 1 .02 | [1.01-1.03] | 0.001 | **1.01** | **[1.00-1.02]** | **0.001** |
| Albumin | 0.95 | [0.86-1.05] | 0.30 |  |  |  |
| Platelets count | 0.97 | [0.98-0.99] | <0.001 | **0.98** | **[0.98-0.99]** | **0.03** |
| Child-Pugh score | 1.50 | [0.93-2.45] | 0.96 |  |  |  |
| ALD | 1.52 | [0.64-3.60] | 0.35 |  |  |  |
| Liver volume index | 1 | [0.99-1.00] | 0.74 |  |  |  |
| Liver / spleen volume ratio | 0.68 | [0.51-0.92] | 0.01 | 0.93 | [0.69-1.26] | 0.65 |
| PPG | 1.20 | [0.99-1.44] | 0.06 |  |  |  |

*ALD, alcohol-related liver disease; PPG, portal pressure gradient*

**Supplementary Table 8 b: Univariate and multivariate analysis of factors associated with overt HE in cohort 1**

|  |  |  |  |  |  |  |
| --- | --- | --- | --- | --- | --- | --- |
|  | Univariate analysis | | | Multivariate analysis | | |
|  | Hazard ratio | IC 95% | p | Hazard ratio | IC 95% | p |
| Gender  Male  Female | 1  0.65 | [0.33-1.29] | 0.22 |  |  |  |
| Age | 1.02 | [0.97-1.06] | 0.49 |  |  |  |
| Total bilirubin (each increase of 1 unit) | 1.01 | [0.99-1.02] | 0.41 |  |  |  |
| Serum creatinine | 1 .02 | [1.01-1.03] | <0.001 | **1.01** | **[1.00-1.01]** | **0.02** |
| Albumin | 0.93 | [0.85-1.02] | 0.11 |  |  |  |
| Platelets count | 0.99 | [0.98-0.99] | 0.02 | 0.99 | [0.99-1.00] | 0.16 |
| Child-Pugh score | 1.03 | [0.70-1.50] | 0.86 |  |  |  |
| Liver volume index | 1 | [0.99-1.00] | 0.57 |  |  |  |
| Liver / spleen volume ratio | 0.79 | [0.63-0.99] | 0.04 | 0.94 | [0.79-1.14] | 0.55 |
| PPG | 1.01 | [0.87-1.18] | 0.89 |  |  |  |
|  |  |  |  |  |  |  |
|  |  |  |  |  |  |  |

*ALD, alcohol-related liver disease; PPG, portal pressure gradient*

**Supplementary Table 8 c: Univariate and multivariate analysis of factors associated with death in cohort 2**

|  | Univariate analysis | | | Multivariate analysis | | |
| --- | --- | --- | --- | --- | --- | --- |
|  | Hazard ratio | IC 95% | p | Hazard ratio | IC 95% | p |
| Age | 1.00 | [0.95-1.06] | 0.95 |  |  |  |
| Total bilirubin (each increase of 1 unit) | 1.02 | [0.99-1.05] | 0.06 |  |  |  |
| Serum creatinine | 0.99 | [0.99-1.01] | 0.65 |  |  |  |
| Albumin | 0.99 | [0.91-1.07] | 0.80 |  |  |  |
| Platelets count | 0.99 | [0.99-1.00] | 0.34 |  |  |  |
| Child-Pugh score | 0.98 | [0.75-1.29] | 0.90 |  |  |  |
| Liver volume index | 0.95 | [0.21-4.34] | 0.95 |  |  |  |
| Liver / spleen volume ratio | 0.95 | [0.72-1.26] | 0.77 |  |  |  |
| PPG | 1.20 | [0.99-1.44] | 0.06 |  |  |  |
|  |  |  |  |  |  |  |
|  |  |  |  |  |  |  |

*ALD, alcohol-related liver disease; PPG, portal pressure gradient*

**Supplementary Table 8 d: Univariate and multivariate analysis of factors associated with overt HE in cohort 2**

|  |  |  |  |  |  |  |
| --- | --- | --- | --- | --- | --- | --- |
|  | Univariate analysis | | | Multivariate analysis | | |
|  | Hazard ratio | IC 95% | p | Hazard ratio | IC 95% | p |
| Age | 1.07 | [1.01-1.13] | 0.01 | 1.08 | 1.02-1.14 | 0.01 |
| Total bilirubin (each increase of 1 unit) | 1.02 | [1.01-1.05] | 0.04 | 1.03 | 1.00-1.05 | 0.01 |
| Serum creatinine | 1.00 | [0.99-1.01] | 0.37 |  |  |  |
| Albumin | 0.98 | [0.92-1.04] | 0.54 |  |  |  |
| Platelets count | 0.99 | [0.99-1.00] | 0.15 |  |  |  |
| Child-Pugh score | 1.30 | [0.98-1.71] | 0.06 |  |  |  |
| Liver volume index | 1 | [1.00-1.00] | 0.45 |  |  |  |
| Liver / spleen volume ratio | 0.92 | [0.75-1.12] | 0.41 |  |  |  |
| PPG | 0.98 | [0.99-1.10] | 0.98 |  |  |  |
|  |  |  |  |  |  |  |
|  |  |  |  |  |  |  |
|  |  |  |  |  |  |  |

*ALD, alcohol-related liver disease; PPG, portal pressure gradient*

**Supplementary Figures**

**Supplementary Figure 1: Acquisition of liver volume**

*
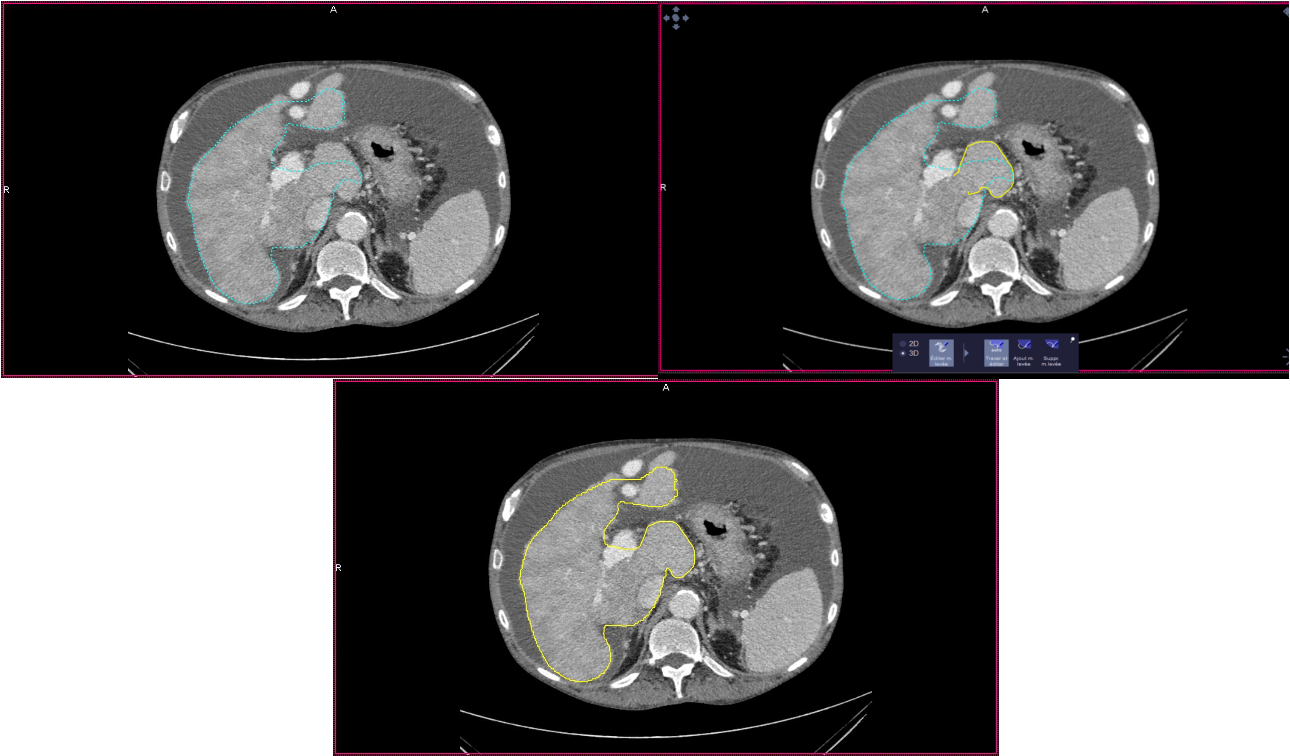
*

a Automatic acquisition of liver contouring; b, manual correction; c, determination of liver volume

## **Supplementary Figure 2: Correlation between standardized liver volume and MELD score**


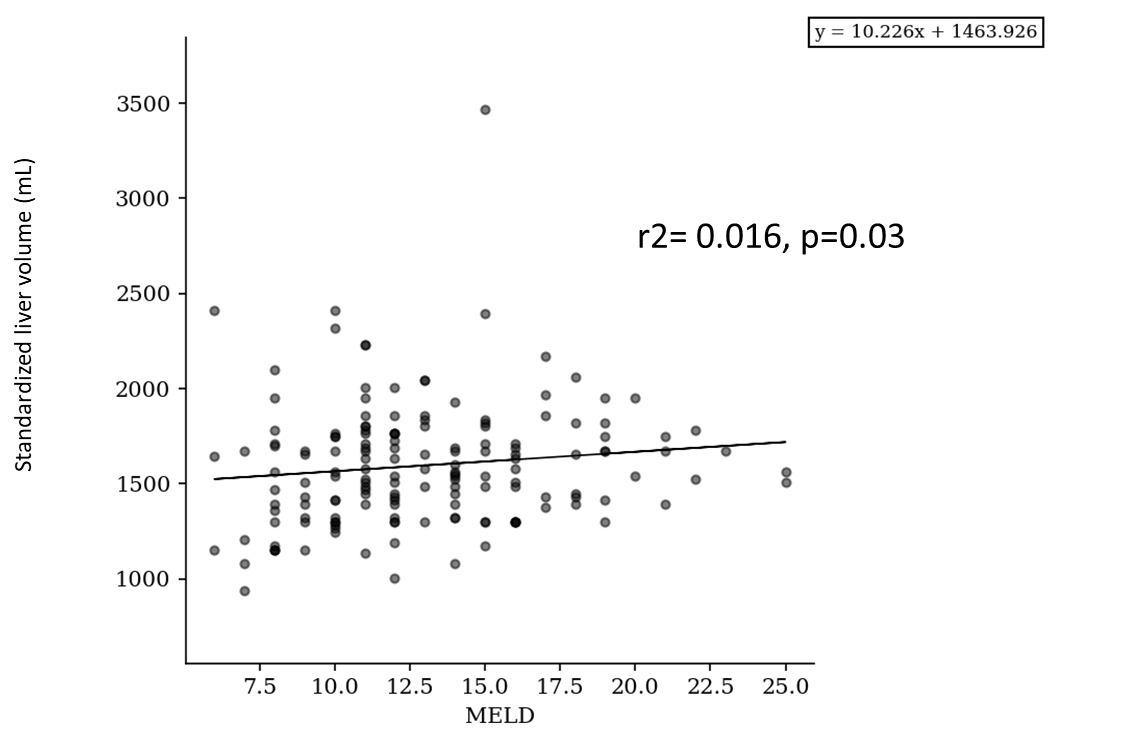


A poor positive correlation was found between standardized liver volume and MELD score (rho= 0.17, r2= 0.016, p=0.03)
